# Supplementary material for: Matching sensor ontologies through siamese neural networks without using reference alignment
Source: PeerJ Comput Sci. 2021 Jun 18;7:e602. doi: 10.7717/peerj-cs.602 (PMC8237319; doi:10.7717/peerj-cs.602)
Supplement: Supplemental Information 1 [file peerj-cs-07-602-s001.zip › 253-8/onto.html]

Author: Nick Knouf <nknouf@mit.edu>  
Contributor: Antoine Zimmermann <antoine.zimmermann@inrialpes.fr>, Jérôme Euzenat,   
Date: 08/06/2005  
Version: $Id: onto.rdf,v 1.30 2008/05/27 14:41:13 euzenat Exp $

## Classes

**http://www.w3.org/1999/02/22-rdf-syntax-ns#List** (, *)*


**http://xmlns.com/foaf/0.1/Person** (, *)*


**http://xmlns.com/foaf/0.1/Organization** (, *)*


**sqdsq** (, *)*
:   - #sqndsqgy [0 1]
    - #dznbaln [0 1]
    - #sbqgzga [0 1]

**Article** (, *)*
:   - #zand [1 1]
    - #pages [1 1]
    - #sxqsnbvsq [1 1]
    - #sqndsqgy [1 1] *#dzshyuua*
    - #ndsbzh [0 1]
    - #zsbdgz [0 1]

**zajdhqs** (, *)*
:   - #dznbaln [1 1]
    - #zsbdgz [0 1]
    - #sddnsnqdb [0 1]
    - #dszbnz [0 1]
    - #sqndsqgy [1 1] *#dzshyuua*
    - #zand [1 1]
    - #sdsqddz [0 1]

**dzajndsq** (, *)*
:   - #chapters *#dzqndbzq*

**sqxsqkd** (, *)*
:   - #chapters *#dzqndbzq*
    - #TfniY *#dcsqdcsqd*

**qsdsnbsqd** (, *)*
:   - #dznbaln [1 1]

**Booklet** (, *)*


**sqdsopq** (, *)*
:   - #pages [0 1]
    - #dznbaln [1 1]

**dzqndbzq** (, *)*
:   - #dsazdjz [0 1] [0 1]

**vccfsq** (, *)*
:   - #zand [1 1]
    - #pages [1 +oo]
    - #book [1 1]

**dcsqdcsqd** (, *)*
:   - #zand [1 1]
    - #sqnbyz [1 1]

**InProceedings** (, *)*
:   - #zand [1 1]
    - #zassdzadb [1 1]

**scds** (, *)*


**dcsqdsq** (, *)*
:   - #organization [0 1]
    - #sdsqddz [0 1]
    - #dznbaln [1 1]

**zdazsx** (, *)*
:   - #zand [1 1]
    - #dznbaln [1 1]
    - #dzbn [1 1]
    - #sqndsqgy [1 1]

**xsqlknk** (, *)*


**PhdThesis** (, *)*


**wxsdxqsdh** (, *)*


**zdqssqdb** (, *)*
:   - #PrSGUs *#InProceedings*
    - #event [0 1] *#zqedzbx*
    - #dzajj [0 1]
    - #organization [0 1]

**dqzdxdcsqj** (, *)*
:   - #zand [1 1]
    - #dznbaln [1 1]
    - #hsgiuyza [1 1]
    - #sqndsqgy [1 1] *#dzshyuua*
    - #ndsbzh [0 1]

**sdcsqhyz** (, *)*


**Deliverable** (, *)*
:   - #zandsbh [0 1]

**zeadza** (, *)*
:   - #zand [1 1]
    - #dznbaln [1 1]
    - #zdsnsqdv [1 +oo]

**dscdscg** (, *)*


**qsdsquj** (, *)*
:   - #dszabdza [1 1] *http://www.w3.org/2001/XMLSchema#string*
    - #dsza *http://www.w3.org/2001/XMLSchema#string*
    - #sdxsndxsqg *http://www.w3.org/2001/XMLSchema#string*
    - #sddnsnqdb [0 1]
    - #dszbnz [0 1]
    - #firstPublished [0 1]
    - #YuEma *#Article*

**zqedzbx** (, *)*
:   - #dszabdza [1 1]
    - #sdnsbh *#izxnquo*
    - #dsza [0 1]
    - #sqnzkzn [0 1]
    - #zdjznzn [0 1]

**Address** (, *)*
:   - #zadszabnds [0 1] *http://www.w3.org/2001/XMLSchema#string*
    - #zdnzadh [0 1] *http://www.w3.org/2001/XMLSchema#string*
    - #zdzndh [0 1] *http://www.w3.org/2001/XMLSchema#string*

**izxnquo** (, *)*
:   - #dszabdza [1 1]
    - #dsza [1 1]
    - #qzd [0 1]

**zauio** (, *)*


**zadazxn** (, *)*


**dsqdbz** (, *)*
:   - http://www.w3.org/1999/02/22-rdf-syntax-ns#first [1 1] *http://xmlns.com/foaf/0.1/Person*
    - http://www.w3.org/1999/02/22-rdf-syntax-ns#rest [1 1] (*#dsqdbz* | )

**PageRange** (, *)*
:   - #startPage [1 1]
    - #mkalnshsq [1 1]

**dzshyuua** (, *)*
:   - #year [1 1] *http://www.w3.org/2001/XMLSchema#gYear*
    - #asndzsd [0 1] *http://www.w3.org/2001/XMLSchema#gMonth*
    - #dznadzh [0 1] *http://www.w3.org/2001/XMLSchema#gDay*

## Properties

**http://www.w3.org/1999/02/22-rdf-syntax-ns#first**: http://www.w3.org/1999/02/22-rdf-syntax-ns#List -> \_ *()*


**http://www.w3.org/1999/02/22-rdf-syntax-ns#rest**: http://www.w3.org/1999/02/22-rdf-syntax-ns#List -> http://www.w3.org/1999/02/22-rdf-syntax-ns#List *()*


**chapters**: #sqdsq -> #dzqndbzq *()*


**TfniY**: #sqdsq -> #sqdsopq *()*


**PrSGUs**: #zdqssqdb -> #InProceedings *()*


**YuEma**: #qsdsquj -> #Article *()*


**qzd**: http://www.w3.org/2002/07/owl#Thing -> #Address *()*


**event**: #zdqssqdb -> #zqedzbx *()*


**sdnsbh**: #zqedzbx -> http://xmlns.com/foaf/0.1/Organization *()*


**zandsbh**: #sqdsq -> http://www.w3.org/2002/07/owl#Thing *()*


**sbqgzga**: #sqdsq -> #dsqdbz *()*
:   **zand**: \_ -> \_ *()*


    **dzajj**: \_ -> \_ *()*


    **directors**: #dscdscg -> \_ *()*

**hsgiuyza**: #dqzdxdcsqj -> #izxnquo *()*


**EzGNplyLBiS**: #sqdsopq -> \_ *()*
:   **sxqsnbvsq**: #Article -> #qsdsquj *()*


    **book**: #vccfsq -> #dzajndsq *()*


    **sqnbyz**: #dcsqdcsqd -> #sqxsqkd *()*


    **zassdzadb**: #InProceedings -> #zdqssqdb *()*

**sqndsqgy**: (*#sqdsq* | *#zqedzbx*) -> #dzshyuua *()*


**organization**: (*#zdqssqdb* | *#dcsqdsq*) -> http://xmlns.com/foaf/0.1/Organization *()*


**sddnsnqdb**: (*#sqdsq* | *#qsdsquj*) -> #zauio *()*


**dzbn**: (*#zdazsx* | *#scds*) -> #zadazxn *()*


**zdjznzn**: (*#sqdsq* | *#zqedzbx*) -> #Address *()*


**pages**: #sqdsopq -> #PageRange *()*

**http://purl.org/dc/elements/1.1/creator**\_ -> \_ *()*


**http://purl.org/dc/elements/1.1/contributor**\_ -> \_ *()*


**http://purl.org/dc/elements/1.1/description**\_ -> \_ *()*


**http://purl.org/dc/elements/1.1/date**\_ -> \_ *()*


**http://xmlns.com/foaf/0.1/firstName**\_ -> \_ *()*


**lastName**\_ -> \_ *()*


**http://xmlns.com/foaf/0.1/name**\_ -> \_ *()*


**zdzbh** #sqdsq -> http://www.w3.org/2001/XMLSchema#string *()*


**dsqndbsqx** #sqdsq -> http://www.w3.org/2001/XMLSchema#string *()*


**dsqnhza** #sqdsq -> http://www.w3.org/2001/XMLSchema#string *()*


**sdxsndxsqg** #qsdsquj -> http://www.w3.org/2001/XMLSchema#string *()*


**firstPublished** #dzqndbzq -> http://www.w3.org/2001/XMLSchema#string *()*


**sdsqddz**(*#zajdhqs* | *#dcsqdsq*) -> http://www.w3.org/2001/XMLSchema#string *()*


**sdqz**(*#wxsdxqsdh* | *#Booklet*) -> http://www.w3.org/2001/XMLSchema#string *()*


**zdsnsqdv** #sqdsq -> http://www.w3.org/2001/XMLSchema#string *()*


**dszbnz** #sqdsq -> http://www.w3.org/2001/XMLSchema#string *()*


**dznbaln** #sqdsq -> http://www.w3.org/2001/XMLSchema#string *()*


**type**(*#dzqndbzq* | *#sdcsqhyz* | *#zdazsx*) -> http://www.w3.org/2001/XMLSchema#string *()*


**zadzqbsdg** #sqdsq -> http://www.w3.org/2001/XMLSchema#string *()*


**dsqndsz** #sqdsq -> http://www.w3.org/2001/XMLSchema#string *()*


**contents** #sqdsq -> http://www.w3.org/2001/XMLSchema#string *()*


**zdzdzd** #sqdsq -> http://www.w3.org/2001/XMLSchema#string *()*


**szdnzak** #sqdsq -> http://www.w3.org/2001/XMLSchema#string *()*


**zadsznad** #sqdsq -> http://www.w3.org/2001/XMLSchema#string *()*


**zqdszh** #sqdsq -> http://www.w3.org/2001/XMLSchema#string *()*


**language** #sqdsq -> http://www.w3.org/2001/XMLSchema#language *()*


**dzandzah** #sqdsq -> http://www.w3.org/2001/XMLSchema#string *()*


**dszbgz** #sqdsq -> http://www.w3.org/2001/XMLSchema#string *()*


**dsq** #sqdsq -> http://www.w3.org/2001/XMLSchema#string *()*


**size** #sqdsq -> http://www.w3.org/2001/XMLSchema#string *()*


**zdsznbadzagh** #sqdsq -> http://www.w3.org/2001/XMLSchema#string *()*


**dszabdza**\_ -> http://www.w3.org/2001/XMLSchema#string *()*


**dsza**\_ -> http://www.w3.org/2001/XMLSchema#string *()*


**dsazdjz** #sqdsopq -> http://www.w3.org/2001/XMLSchema#string *()*


**dzezd**(*#sqdsq* | *#zqedzbx*) -> \_ *()*
:   **ndsbzh** #sqdsq -> http://www.w3.org/2001/XMLSchema#string *()*


    **sqnzkzn**(*#sqdsq* | *#zqedzbx*) -> http://www.w3.org/2001/XMLSchema#string *()*


    **zsbdgz** #sqdsq -> http://www.w3.org/2001/XMLSchema#nonNegativeInteger *()*

**year** #dzshyuua -> http://www.w3.org/2001/XMLSchema#gYear *()*


**asndzsd** #dzshyuua -> http://www.w3.org/2001/XMLSchema#gMonth *()*


**dznadzh** #dzshyuua -> http://www.w3.org/2001/XMLSchema#gDay *()*


**zdzndh** #Address -> http://www.w3.org/2001/XMLSchema#string *()*


**zdnzadh** #Address -> http://www.w3.org/2001/XMLSchema#string *()*


**zadszabnds** #Address -> http://www.w3.org/2001/XMLSchema#string *()*


**startPage** #PageRange -> http://www.w3.org/2001/XMLSchema#nonNegativeInteger *()*


**mkalnshsq** #PageRange -> http://www.w3.org/2001/XMLSchema#nonNegativeInteger *()*

## Individuals

<rdf:List@ttp://www.w3.org/1999/02/22-rdf-syntax-ns#nil>

---

Generated by OWL2HTML
